# Reliability of a low-cost webcam recording system for three-dimensional lower limb gait analysis

**DOI:** 10.1080/23335432.2019.1671221

**Published:** 2019-09-26

**Authors:** Apiwan Pusara, Sumet Heamawatanachai, Komsak Sinsurin, Chaiyong Jorrakate

**Affiliations:** a Faculty of Allied Health Sciences, Naresuan University, Phitsanulok, Thailand; b Faculty of Engineering, Naresuan University, Phitsanulok, Thailand; c Biomechanics and Sports Research unit, Faculty of Physical Therapy, Mahidol University, Nakhon Pathom, Thailand

**Keywords:** Low-cost, reliability, webcam, gait, three-dimensional

## Abstract

The purposes of this study were to develop and evaluate the test-retest reliability of a specific low-cost three-dimensional webcam recording system (3D-WCRS) and compare its reliability to a standard motion analysis system. Twenty healthy volunteers comprised of 5 males and 15 females with a mean age of 22.90 years and mean BMI of 22.72 kg/m^2^ were investigated for angles of hip, knee and ankle joints in three planes while walking at a self-selected speed. Intraclass correlation coefficients (ICCs) were used to evaluate as well as compare the test-retest reliability of the 3D-WCRS and standard motion analysis system. Standard error of measurement (SEM) was also analyzed for the purposes of the study. The results exhibited excellent test-retest reliability for the 3D-WCRS (ICCs ranged between 0.93 and 0.99, p = 0.001) in the three joints and planes. The standard motion analysis system demonstrated excellent reliability for all joints and planes (ICCs ranged between 0.99 and 1.00, p = 0.001). Minimal SEM values were observed in both the 3D-WCRS and standard motion analysis systems. Therefore, the developed low-cost 3D-WCRS exhibits good to excellent test-retest reliability. The test-retest reliability of the 3D-WCRS is likely to be comparable to a standard motion analysis system.

## Introduction

Walking is an essential human activity for daily living (Kirtley 2006). If a person’s gait is abnormal, it will affect their quality of life and may also result in high expenditure for rehabilitation. To evaluate abnormal gait patterns, a gait analysis measurement system is an important tool for the specific treatment planning of each patient (Barak et al. 2006; Kirtley 2006). Presently, significant evidence exists to suggest that clinicians typically assess the gait of patients through visual observation due to a lack of necessary equipment, as well as time consumption and financial support (Eastlack et al. 1991; Coutts 1999; Watelain et al. 2005; Williams et al. 2009; Carse et al. 2013). However, visual observation alone is not reliable and has low accuracy with disagreement in the descriptive terminology used for gait analysis among observers (Watelain et al. 2005; Williams et al. 2009). Observers may not be able to accurately memorize the gait patterns of patients because of the complexity of the motions.

A camera-based three-dimensional (3D) motion analysis system has been developed to assess the complexity of kinematic gait parameters such as joint angles, joint position, body alignment, and gait speed. The advantages of this system are accuracy, reliability, and providing a range of gait parameters within one analysis (Simon 2004; Miller and Callister 2009). While 3D motion analysis systems have been widely used in laboratory research, they are rarely used in clinical settings due to limitations including the expense of the system, sophisticated tool kits, time-consuming processes, and the need for specialists.

Nowadays, a webcam is efficacious to use instead of an expensive camera (Simon 2004). Wang et al. (2013) developed a low-cost video system in 2013 to assess the gait of elderly people. Their results showed that the developed low-cost system was able to assess spatiotemporal gait parameters in the elderly; however, the joint angles of lower extremities were not measured in their study. In 2010, Kongkhiaw (2010) developed a real-time gait assessment tool with a webcam recording system. This novel system showed reliable output for lower extremity joint angles. Nevertheless, the motion system from this study performed two-dimensional analysis only. Therefore, the current study aims to evaluate the test-retest reliability of a low-cost three-dimensional webcam recording system (3D-WCRS) compared to the standard 3D motion analysis system. The current study intends to develop the 3D-WCRS to reliably measure the joint angles for hips, knees and ankles. This developed low-cost 3D motion analysis system might be promoted for future use in clinical settings.

## Materials and methods

### Participants

Twenty healthy volunteers (5 males, 15 females, mean age of 22.90 ± 2.20 years, BMI of 22.72 ± 3.38 kg/m^2^) were recruited in this study using convenience sampling. Inclusion criteria were individuals aged between 21 and 40 years who were healthy and had no deformities or abnormalities of the joints in the lower limbs, such as bowed legs, club foot or other obvious and debilitating deformities. Participants were excluded if they had suffered any major orthopedic injury, such as a meniscus tear, ligament tear, and/or recent muscle strain or tendon sprain, fracture or any surgery to the lower extremities within the last year before participating in this study. In addition, participants were excluded if they had recently experienced abnormal orthopedic conditions, such as hip pain or knee osteoarthritis and/or neuromuscular deficits which could considerably affect their gait. Pregnant or menstruating females were likewise excluded. The experimental protocol was approved by the Naresuan University Institutional Review Board (IRB No. 0616/60). Participants signed a written informed consent before joining the tests.

### Instruments

#### 3D-WCRS

**Figure 1.**
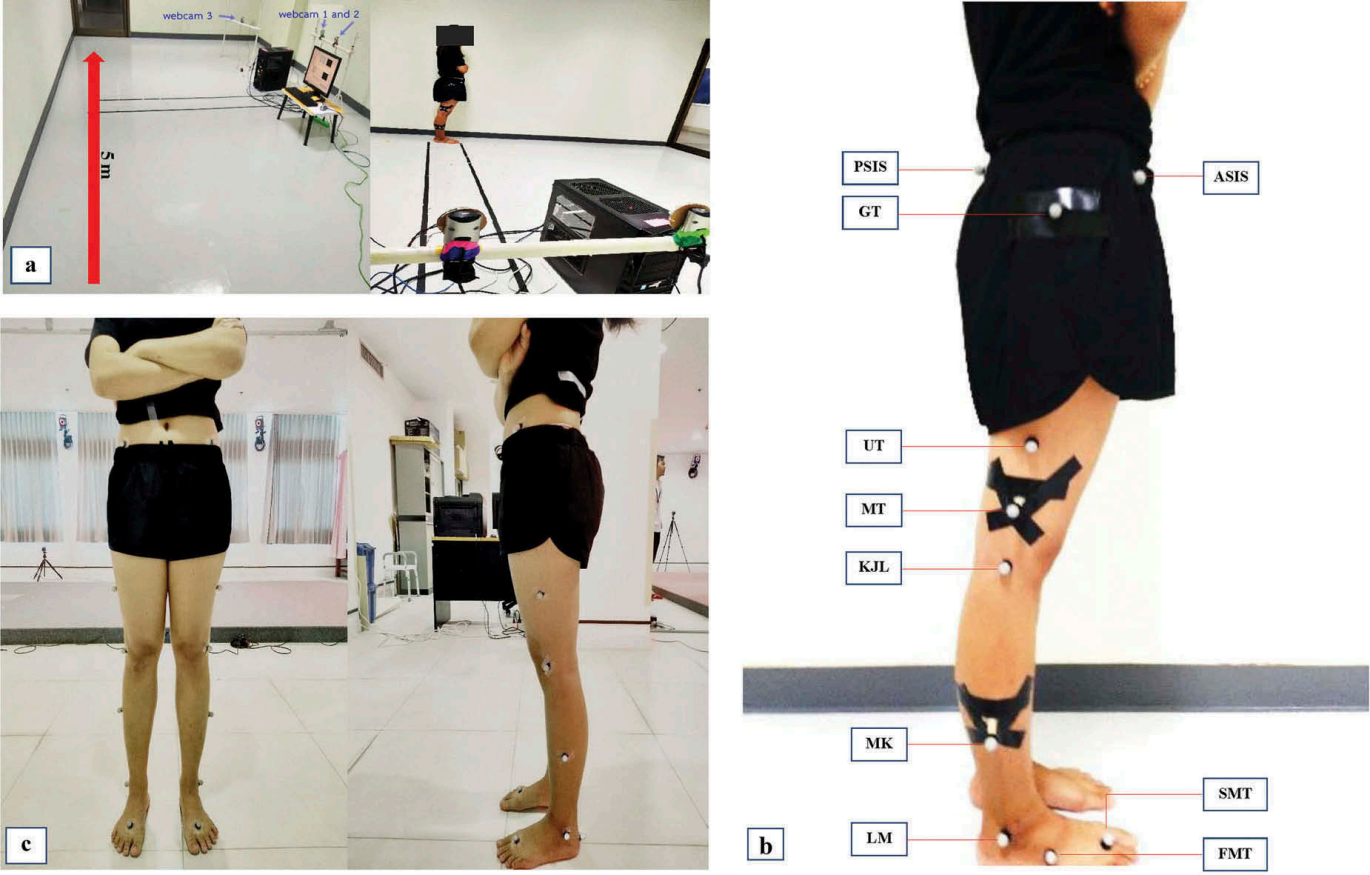
Demonstration of laboratory setting for 3-DWCRS. (b). Positions for marker placement in lower limbs under the reliability testing of 3D-WCRS (PSIS = posterior superior iliac spine, ASIS = anterior inferior iliac spine, GT = greater trochanter, UT = upper thigh, MT = mid-thigh, KJL = knee joint line, MK = mid-knee, LM = lateral malleolus, FMT = base of fifth metatarsal bone and SMT = second head of metatarsal bone). (C). Positions for marker placement in lower limbs for a standard 3D motion analysis system.

The developed 3D-WCRS consisted of three webcams (OKER, 2.0 MP, 30 frames per second; fps and infrared), a personal computer (LG Intel® Core™ i5-470, CPU 3.20 GHz), reference frame, 14 mm spherical markers, tape rule and tripod kits. The software for calculating joint motion in this study was developed by a researcher with LabVIEW and NI Vision development module. The developed software extracted images from three video files that were previously recorded by three webcams simultaneously. The software transformed the extracted images into Hue Saturation Luminance (HSL) color format. Then, the sets of pixels of markers in the extracted images were obtained using image segmentation. The ranges of HSL values were set as H (0–255), S (232–255) and L (0–46) respectively. After that, the center pixels of markers of images from cameras were used to calculate their positions in a Cartesian co-ordinate system (x, y, z) using stereo-vision. The stereo-vision technique is used for processing the inputs from digital images from more than two cameras. It matches pixels according to the principle of triangulation and constructs a three-dimensional estimation of an object’s position, including length, width and depth (Bebeselea-Sterp et al. 2017). The joint angles (hip, knee and ankle angles) were calculated by the co-ordinates of the related markers of the joints. Because of the limitations of laboratory space and 3D-WCRS camera setting, only the angles of joint motion in right lower limbs were selectively analyzed in the current study (Figure 1(b)).

Prior to collecting the data, the 3D-WCRS was calibrated with a reference frame with reflective markers. The reference frame consisted of a rectangular chart (60 × 60 cm) with nine reflective (14-mm diameter) markers with known x, y, z co-ordinate positions. The reference frame was put in the middle of the capture area and was seen clearly by three webcams. The calibration was analyzed by determining differences between the x, y, z co-ordinate positions of all markers between the reference frame and the recorded values from the 3D-WCRS. The calibration was accepted when the averaged errors of the x, y, z co-ordinate positions from all markers were less than 5% of the actual positions.

#### The standard motion analysis system: Vicon^TM^ motion analysis system

The standard motion analysis system referenced in the current study is the Vicon^TM^ Motion Analysis System. This system consists of a 10-camera Vicon™ Nexus system (Oxford Metrics, Oxford, UK) at 200 Hz, a personal computer, calibration kits, and reflective spherical markers. Plug-in Gait Model software (Oxford Metrics, Oxford, England) was used to construct a three-dimensional model of lower extremities (Dempster 1955). Only right hip, knee and ankle joint angles of participants were analyzed in the gait cycle. Kinematic data were filtered with a 4th order zero-lag low-pass Butterworth filter at 6 Hz.

### Experimental procedure

#### Test-retest reliability of 3D-WCRS system

Prior to the start of the study, the researchers gained experience in the use of the 3D-WCRS, the standard motion analysis system and the method of marker attachment. Researchers initially collected participant joint motion in the right hip, knee and ankle joints at the Faculty of Engineering, Naresuan University. The laboratory settings are detailed in Figure 1(a). The participants were assessed for hip, knee and ankle joint angles for the test-retest reliability of the 3D-WCRS for two sessions. On the test date, participants wore a black fitted t-shirt and shorts during testing in the laboratory. Next, a set of 14 mm-diameter spherical reflective markers were attached with adhesive tape on the participants’ bony landmarks, including the anterior superior iliac spine (ASIS), posterior superior iliac spine (PSIS), greater trochanter, lateral aspect of the mid-thigh, middle of the lateral knee joint line, lateral malleolus, base of the fifth metatarsal bone, and head of the second metatarsal bone. Five-centimeter wand markers were placed on the lateral sides of the mid-thigh and shank (Figure 1(b)). The maker set used in the 3D-WCRS was designed to represent the segments of the lower limb. The joint angles were calculated from one segment relative to nearby segments and also calculated in relation to the joint angles in static trial. The wand markers were used to calculate joint angles in the horizontal plane. After obtaining the marker locations, the joint angles were calculated using Microsoft Excel function as follows:

In sagittal plane; hip angle = ATAN2(X_KJL –_ X_UT_, Y_KJL –_ Y_UT_), knee angle = ATAN2(X_LM –_ X_KJL_, Y_LM_ – Y_KJL_) – ATAN2(X_KJL –_ X_UT_, Y_KJL_ – Y_UT_), and ankle angle = ATAN2(X_SMT –_ X_LM_, Y_SMT –_ Y_LM_) – ATAN2(X_LM –_ X_KJL_, Y_LM –_ Y_KJL_).

In frontal plane; hip angle = ATAN2(Z_KJL –_ Z_UT_, Y_KJL_ -Y_UT_), knee angle = ATAN2(Z_LM_ – Z_KJL_, Y_LM_ – Y_KJL_) – ATAN2(Z_KJL –_ Z_UT_, Y_KJL_ -Y_UT_), and ankle angle = ATAN2(Z_FMT –_ Z_LM_, Y_FMT_ – Y_LM_) – ATAN2(Z_LM –_ Z_KJL_, Y_LM –_ Y_KJL_).

In horizontal plane, hip angle = ATAN2(Z_MT –_ Z_MT1_, X_MT_ – X_MT1_), knee angle = ATAN2(Z_MK_ – Z_MK1_, X_MK_ – X_MK1_) – ATAN2(Z_MT –_ Z_MT1_, X_MT_ – X_MT1_), and ankle angle = ATAN ((Z_SMT_ – Z_LM_) /((X_SMT_ – X_LM_)^2^+(Y_SMT_ – Y_LM_)^2^)°^5^).

Where; X, Y and Z were co-ordinates, and the letters following the co-ordinates were the marker name (Figure 1(b)).

Later, participants practiced walking along a specific test path to gain familiarity before the recording of data. In the actual tests, participants were initially recorded from the static trial in an upright standing position, which was used for initial reference angles when analyzing hip, knee and ankle joints during walking. Afterward, individual participants were assessed for gait and recorded using the 3D-WCRS while walking at their natural self-selected speed along the test path (5 m) for 10 trials. A day after the first session, gait assessments with 3D-WCRS were recollected for all participants in a second session. Lastly, the researchers manually digitized and tracked the exported information of the gait cycle including hip, knee and ankle joint angles.

#### Test-retest reliability of the standard three-dimensional motion analysis system

When data collection at Naresuan University was completed, participants were appointed to test again at another laboratory at the Faculty of Physical Therapy, Mahidol University, to investigate the test-retest reliability of the standard motion analysis system. First, the standard motion analysis system was calibrated, followed by measurement of weight, height, leg length, knee width, and ankle width for individual participants before data collection. Individual participants had spherical reflective markers (14 mm in diameter) attached on their bony landmarks with adhesive tape according to the modified Helen Hayes marker set (Christopher 2006). The locations of marker placement were bilateral ASIS and PSIS, lateral aspects of the thighs, lateral condyle of the femurs, mid-tibias, lateral malleoli, and the heels and heads of the second metatarsal bones (Figure 1(c)). Afterward, participants practiced walking along the specific test path to gain familiarity before the recording of data. Then, participants were also recorded starting from a standing position. Next, individual participants were recorded for their gait using the standard motion analysis system while walking at their natural self-selected speed along the testing path (10 m) for 10 trials. Lastly, Plug-in Gait was used to calculate the angles of the lower limbs during the gait cycle.

### Data acquisition and statistical analysis

Hip, knee and ankle joint angles during eight phases of the gait cycle of the 20 participants were calculated by the same researcher from 3D-WCRS and the standard motion analysis system using the same criteria for data analysis as follows. The researcher selected five successful trials which showed a complete gait cycle and no loss of markers in the recorded pictures. The selected pictures of the gait cycle were separately analyzed into eight events including heel strike, foot flat, midstance, heel off, toe off, initial swing, mid-swing and late swing phases by visual inspection (Nordin and Frankel 2012). Heel strike was the picture where the heel initially contacts the floor. Foot flat was the picture showing the whole plantar surface placed on the floor. Mid stance was the picture where the whole body weight was loaded on the leg and the position of the lower leg was vertically arranged to the floor. Heel off and toe off were defined when the heel and the toe were initially lifted from the floor, respectively. The picture of the initial swing was selected from the middle picture recorded between toe off and mid-swing phases. The picture of mid-swing was chosen if the analyzed leg was under the midline of the body. The picture of late swing was picked out from the middle picture between mid-swing and a second heel contact phases. The researcher randomly selected successful gait trials if there were more than five successful trials. In the manual digitization process, the researcher visually selected the pictures of each phase of the gait cycle and transferred the x, y, z co-ordinate positions from the recorded picture to calculate the joint angle relatively to the static trial. Mean, standard deviation (SD) and standard error of measurement (SEM) for hip, knee and ankle joint angles were averaged from five trials. There were 160 gait data samples (8 phases × 20 participants) per session for each joint and plane.

Intraclass correlation coefficient (2-way mixed effects, absolute agreement) was used to analyze the test-retest reliability of the 3D-WCRS and the standard motion analysis system. Test-retest reliability was analyzed with a single joint angle across five trials within each session, whereas the averaged joint angle was used to determine test-retest reliability between sessions. ICC values were identified as excellent (ICC > 0.90), good (ICC between 0.75 and 0.90), moderate (0.50–0.75) and poor (ICC < 0.50) reliability (Portney and Watkins 2009). Average walking speeds over the gait cycle were manually calculated after data collection for both measurement systems using right ASIS marker, measured in the direction that volunteers traveled. One-way repeated measures with a Bonferroni’s post hoc analysis were used to determine the differences between average walking speeds between session 1 (from the 3D-WCRS), session 2 (from the 3D-WCRS) and the standard 3D motion analysis system. Moreover, the mean differences of joint angles between the 3D-WCRS and the standard motion analysis system were also descriptively reported. Statistical analyses were performed using SPSS statistical software and a p-value of 0.05 was set for all statistical analyses.

## Results

The test-retest reliability of both within and between sessions of the 3D-WCRS expressed as ICC is provided in Table 1. The ICC values of both within and between sessions were good to excellent for each plane of hip, knee and ankle joints (ICC of first session = 0.89–0.99, ICC of second session = 0.86–0.99, p = 0.01). Test-retest reliability between the first and second sessions was excellent for all joints and planes (ICC = 0.93–0.99, p = 0.01). The test-retest reliability in a single session of the standard 3D motion analysis system expressed as ICCs is provided in Table 2. The ICC values were excellent for all joints and planes (ICC = 0.99–1.00, p = 0.01). The joint angles (mean ± SD) obtained by the 3D-WCRS are presented in the supplementary information.

**Table 1.** Test-retest reliability coefficients of the 3D-WCRS within and between sessions (n = 160).

	ICCs (95% confidence interval)								
			between trials						
	1^st^ session	2^nd^ session	1^st^ session	2^nd^ session	1^st^ session	2^nd^ session	between sessions		
joints	sagittal		frontal		horizontal		sagittal	frontal	horizontal
hip	0.99*	0.99*	0.90*	0.92*	0.97*	0.97*	0.99*	0.94*	0.97*
	(0.99 – 1.00)	(0.99 – 1.00)	(0.87 – 0.92)	(0.89 – 0.93)	(0.96 – 0.97)	(0.96 – 0.97)	(0.99 – 1.00)	(0.93 – 0.95)	(0.97 – 0.98)
knee	0.99*	0.99*	0.89*	0.86*	0.96*	0.97*	0.99*	0.93*	0.96*
	(0.99 – 1.00)	(0.99 – 0.994)	(0.86 – 0.91)	(0.83 – 0.89)	(0.95 – 0.97)	(0.96 – 0.97)	(0.99 – 1.00)	(0.92 – 0.95)	(0.95 – 0.97)
ankle	0.98*	0.98*	0.97*	0.97*	0.97*	0.97*	0.98*	0.97*	0.97*
	(0.98 – 0.99)	(0.97 – 0.98)	(0.96 – 0.97)	(0.96 – 0.97)	(0.96 – 0.98)	(0.96 – 0.97)	(0.97 – 0.98)	(0.96 – 0.98)	(0.96 – 0.98)

ICCs: intraclass correlation coefficients; *p-value = 0.01

**Table 2.** Test-retest reliability coefficients for hip, knee and ankle joints in three planes obtained by standard 3D motion analysis system (n = 160).

ICCs (95% confidence interval)			
	joints		
planes	hip	knee	ankle
sagittal	0.99*	0.99*	0.99*
	(0.99 – 1.00)	(0.99 – 1.00)	(0.99 – 1.00)
frontal	0.99*	0.99*	0.99*
	(0.99 – 1.00)	(0.99 – 1.00)	(0.99 – 1.00)
horizontal	0.99*	0.99*	0.99*
	(0.99 – 1.00)	(0.99 – 1.00)	(0.99 – 1.00)

ICCs: intraclass correlation coefficients; *p-value = 0.01.

The averages of SEM for hip, knee and ankle joints from eight consecutive phases during the gait cycle in three planes as evaluated by the 3D-WCRS and the standard 3D motion analysis system are presented in Table 3. The SEM values for hip, knee and ankle joints in the sagittal plane were minimal (SEM of the first session ranged between 0.09º and 0.83º; 0.19º and 2.33º; 0.11º and 1.52º and SEM of the second session ranged between 0.43º and 2.02º; 0.43º and 2.76º; 0.22º and 2.31º). The SEM values for hip, knee and ankle joints in the frontal (SEM of the first session ranged between 0.28º and 1.42º; 0.08º and 1.33º; 0.39º and 2.03º and SEM of the second session ranged between 0.25º and 1.50º; 0.23º and 5.60º; 0.38º and 1.90º) and horizontal planes (SEM of the first session ranged between 0.35º and 1.45º; 0.55º and 2.99º; 0.30º and 3.10º and SEM of the second session ranged between 0.20º and 1.48º; 0.43º and 1.98º; 0.26º and 1.91º) were also minimal. In addition, smaller SEM values from the standard 3D motion analysis system were observed (SEM of hip, knee and ankle joints in three planes ranged between 0.08º and 0.86º; 0.03º and 0.28º; 0.10º and 0.45º).

**Table 3.** Means and standard deviations for standard error of measurement (SEM) obtained by 3D-WCRS and standard 3D motion analysis system of eight consecutive phases during a gait cycle in three planes (n = 20).

	Hip joint						Knee joint							Ankle joint				
	3D-WCRS						3D-WCRS						3D-WCRS					
	1st session		2nd session		Standard 3D motion system		1st session		2nd session		Standard 3D motion system		1st session		2nd session		Standard 3D motion system	
Planes	Mean	SD	Mean	SD	Mean	SD	Mean	SD	Mean	SD	Mean	SD	Mean	SD	Mean	SD	Mean	SD
Sagittal	0.38°	0.25^o^	0.35^o^	0.22^o^	0.16^o^	0.08^o^	0.84^o^	0.86^o^	0.99^o^	0.89^o^	0.31^o^	0.16^o^	0.42^o^	0.49^o^	0.74^o^	0.74^o^	0.36^o^	0.25^o^
Frontal	0.76^o^	0.37^o^	0.72^o^	0.46^o^	0.11^o^	0.0 7^o^	0.60^o^	0.4 6^o^	1.81^o^	1.78^o^	0.10^o^	0.09^o^	0.93^o^	0.55^o^	0.93^o^	0.47^o^	0.05^o^	0.01^o^
Horizontal	0.69^o^	0.39^o^	0.69^o^	0.45^o^	0.19^o^	0.07^o^	1.27^o^	0.77^o^	1.07^o^	0.46^o^	0.16^o^	0.02^o^	0.81^o^	0.97^o^	0.67^o^	0.59^o^	0.22^o^	0.10^o^

**Figure 2.**
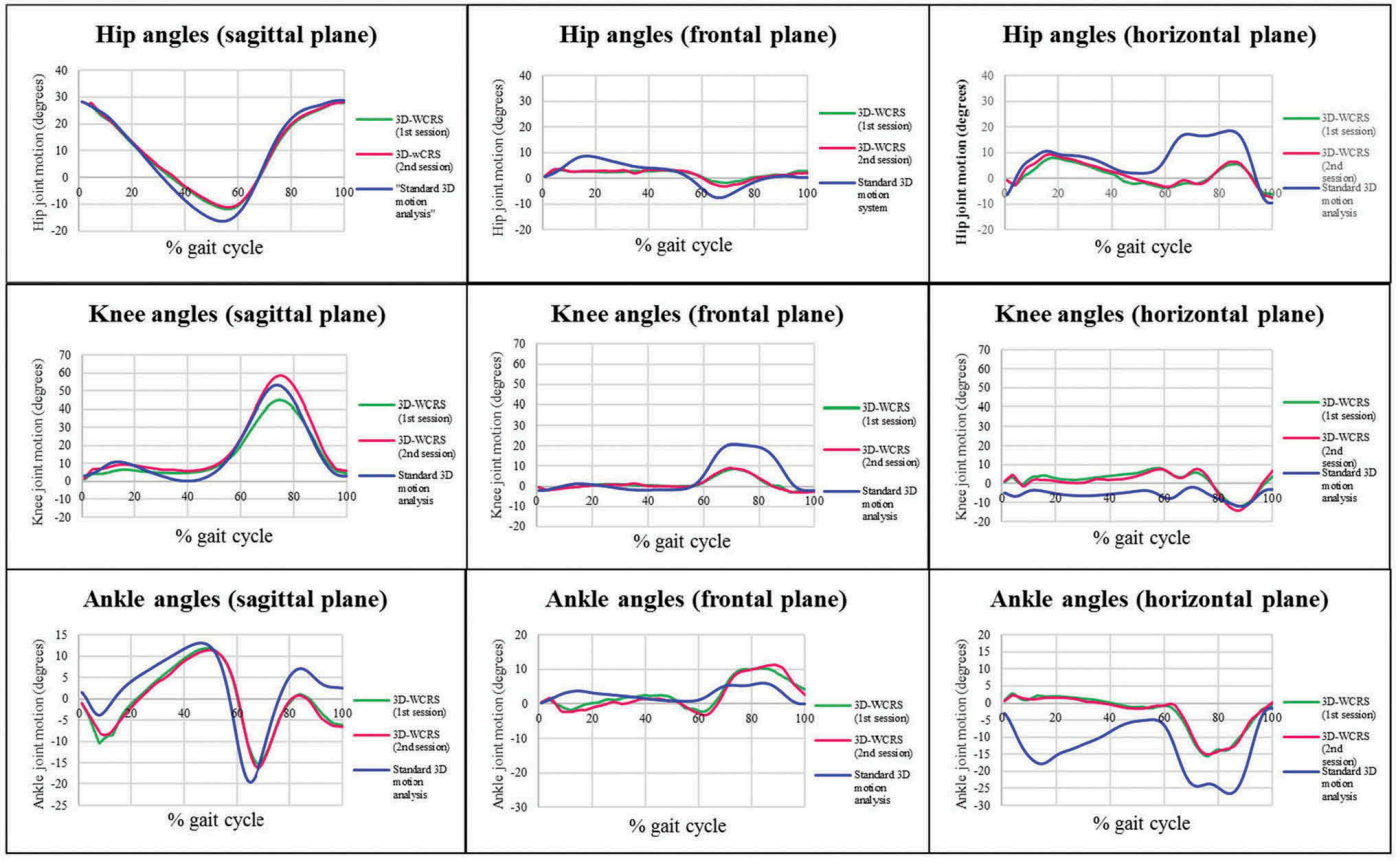
Trajectory of average hip, knee and ankle joint motions in three planes during 100% gait cycle analyzed using the 3D-WCRS (session 1 and 2) and a standard motion analysis system.

The trajectory of hip, knee and ankle joint motions in three planes of 3D-WCRS against the standard motion analysis system were plotted and are shown in Figure 2. Most of the joint trajectory patterns obtained by 3D-WCRS looked similar to the motions observed by the standard motion analysis system, especially in the sagittal plane. The average walking speeds from 3D-WCRS were 0.89 ± 0.08 m/s (session 1), 0.92 ± 0.10 m/s (session 2) and the average walking speed from the standard 3D motion analysis system was 1.21 ± 0.08 m/s. Average walking speeds were significantly different (F_(2, 28)_ = 133.86, p = 0.001) between the 3D-WCRS and the standard 3D motion analysis system in session 1 (mean difference = 0.32 m/s, p = 0.001) and session 2 (mean difference = 0.29 m/s, p = 0.001). There was no difference in average walking speeds between session 1 and 2 with the 3D-WCRS (mean difference = −0.03 m/s, p = 0.44). Means, minimums and maximums of differences of joint angles in the hip, knee and ankle between 3D-WCRS and the standard 3D motion analysis system are shown in Table 4. The average joint angle differences were smaller for all joints in the sagittal and frontal planes when compared with those angles in the horizontal plane.

**Table 4.** Means, minimums and maximums for mean differences in hip, knee and ankle joint angles between 3D-WCRS and standard 3D motion analysis system of eight consecutive phases during a gait cycle in three planes (n = 20).

	3D-WCRS versus standard 3D motion system					
	hip joint		knee joint		ankle joint	
	1^st^ session	2^nd^ session	1^st^ session	2^nd^ session	1^st^ session	2^nd^ session
planes	mean(min, max)	mean(min, max)	mean(min, max)	mean(min, max)	mean(min, max)	mean(min, max)
sagittal	1.16^o^	1.65 ^o^	3.71 ^o^	4.08 ^o^	-2.92 ^o^	-3.52 ^o^
	(-24.41^o^, 20.73°)	(-22.01^o^, 22.38°)	(-19.38^o^, 22.77°)	(-21.45^o^, 26.88°)	(-23.82^o^, 19.42°)	(-16.86 ^o^, 14.85°)
frontal	0.94^o^	1.02^o^	-3.48^o^	-3.92^o^	1.15^o^	0.71^o^
	(-11.34°, 13.70^o^)	(-10.25°, 13.26^o^)	(-39.61°, 14.53^o^)	(-40.49°, 14.48^o^)	(-26.84°, 35.96^o^)	(-28.20°, 42.31^o^)
horizontal	-6.64^o^	-6.72^o^	7.77^o^	7.11^o^	9.21^o^	8.57^o^
	(-57.87°, 24.58^o^)	(-48.77°, 28.46^o^)	(-26.50°, 42.18^o^)	(-33.77°, 39.82^o^)	(-28.50°, 52.05^o^)	(-29.08°, 50.52^o^)

## Discussion

The aims of this study were to evaluate the test-retest reliability of a low-cost 3D-WCRS, both within and between sessions, and to compare its reliability to a standard 3D motion analysis system.

Generally, the results demonstrated that the test-retest reliability coefficients, both within and between sessions of the 3D-WCRS, were good to excellent for all joints and planes. Additionally, the SEM values observed by both the 3D-WCRS and the standard motion analysis system were found to be low (<5º) (HCW et al. 2006; Macionis 2013). Moreover, the trajectories for lower limb motion during the gait cycle from the 3D-WCRS and the standard motion analysis system were similar. This indicated that the developed 3D-WCRS is adequately reliable for determining hip, knee and ankle joint angles during gait in three planes. Therefore, the developed 3D-WCRS is likely to be as reliable as the standard 3D motion analysis system. Although the ranges of mean differences of joint angles during walking between the 3D-WCRS and the standard 3D motion analysis system were large, these differences would not be suitable for representing the similarity between the two measurement systems as measurements of joint motions taken by the two systems were taken on different days and in different places.

The SEM of knee and ankle joints in all planes during toe off, initial swing and mid-swing phases in both sessions were larger as above quantify when compared with the hip joint and other phases. The decreased recording frame rate of the 3D-WCRS may have caused some variability. It was possible that motions of knee and ankle in these phases were rapidly moved and some of the captured pictures from webcams were obscured. This means that the webcams could not effectively record rapid motions. During the recording of data, the decreasing of frame rate for webcams was observed (from 30 fps to approximately 20 fps). This was one limitation of the 3D-WCRS in that frame rate of the webcams was automatically lowered while simultaneously recording motion. Accordingly, the number of webcam frame rates would be a source of variability in terms of joint angles in those phases. In addition, the inconsistency of walking speed among participants may cause variability in measurements. Although participants may already have been familiar with the specific test path before data collection and asked to walk at their natural speed, it could not be assured whether participants walked with a consistent speed. The results demonstrated that walking speeds from the 3D-WCRS (in session 1 and 2) were significantly different from the standard 3D motion analysis system. When the walking speed of individual participants was different, it affected their gait patterns and created inconsistency (McGinley et al. 2009; Kwon et al. 2015).

Joint angle errors between trials in the current study were similarly observed in previous studies which demonstrated variability in motion analysis measurement. A systematic review of McGinley et al. in 2009 (2009) reported that most studies which measured joint angles of the lower limbs during walking by three-dimensional gait analysis systems demonstrated small errors (<4°) of joint angles between trials except the angles in hip and knee rotation. Fosang et al. (2003) and McDowell et al. (2000) also found that the variation of flexion and extension angles in the lower limbs could range from 5 to 10 degrees. The range of SEM observed with the 3D-WCRS ranged within 5 degrees. It could be said that the variability from 3D-WCRS is acceptable when compared to the standard 3D motion analysis system.

The 3D-WCRS was deliberately developed to be a low-cost motion analysis system for convenient use in clinical settings. The 3D-WCRS was preliminarily established as a prototype recording system. The cost of equipment and software for developing the 3D-WCRS was quite low (approximately 1,500 US dollars) when compared to the standard 3D motion analysis system (at least 60,000 US dollars) (Carse et al. 2013). However, there were some limitations in the current study. As a prototype, the 3D-WCRS cannot report in real-time for joint angle or gait speed. Furthermore, it entailed time-consuming manual analysis, visual inspection of gait in different phases and only one side of the lower limb could be measured due to a restriction in the field of view. In addition, the frame rates for webcams were decreased during recording and time-consumption as well as skill-dependence for calculating data was high. These limitations could be solved and evolved in a subsequent study. In future studies, the 3D-WCRS should synchronously investigate gait analysis with the standard motion analysis system to prove its concurrent validity. Furthermore, participants with abnormal gait should be included for investigating the performance of the 3D-WCRS, such as individuals with osteoarthritis and neuromuscular deficits. In conclusion, the 3D-WCRS exhibited mostly good to excellent test-retest reliability and is likely to be as reliable as a standard 3D motion analysis system but at a much lower cost. The 3D-WCRS is a more cost-effective recording system when compared to a standard 3D motion analysis system and can reliably analyze the angles of the lower limbs in three dimensions during walking.

## Supplementary Material

Supplemental Material

## References

[CIT0001] Barak Y, Wagenaar RC, Holt K. 2006 Gait characteristics of elderly people with a history of falls: a dynamic approach. Phys Ther. 86(11):1501–1510. 17079750 10.2522/ptj.20050387

[CIT0002] Bebeselea-Sterp E, Brad R, Brad R. 2017 A comparative study of stereovision algorithms. Int J Adv Comput Sci Appl. 8(11):359–375.

[CIT0003] Carse B, Meadows B, Bowers R, Rowe P 2013 Affordable clinical gait analysis: an assessment of the marker tracking accuracy of a new low-cost optical 3D motion analysis system. Physiotherapy. 99(4):347–351. 23747027 10.1016/j.physio.2013.03.001

[CIT0004] Christopher K 2006 Clinical gait analysis: theory and practice. 1st ed. USA: Elsevier Health Sciences.

[CIT0005] Coutts F 1999 Gait analysis in the therapeutic environment. Man Ther. 4(1):2–10. 10463015 10.1016/s1356-689x(99)80003-4

[CIT0006] Dempster WT 1955 Space requirements of the seated operator. Ohio: Wright-Patterson Air Force Base WADC Technical Report 55–159.

[CIT0007] Eastlack ME, Arvidson J, Snyder-Mackler L, Danoff JV, McGarvey CL 1991 Interrater reliability of videotaped observational gait-analysis assessments. Phys Ther. 71(6):465–472. 2034709 10.1093/ptj/71.6.465

[CIT0008] Fosang AL, Galea MP, McCoy AT, Reddihough DS, Story I 2003 Measures of muscle and joint performance in the lower limb of children with cerebral palsy. Dev Med Child Neurol. 45(10):664–670. 14515937 10.1017/s0012162203001245

[CIT0009] HCW DV, Terwee CB, Knol DL, Bouter LM 2006 When to use agreement versus reliability measures. J Clin Epidermiol. 59:1033–1039. 10.1016/j.jclinepi.2005.10.015 16980142

[CIT0010] Kirtley C 2006 Clinical gait analysis: theory and practice. New York (NY): Churchill Livingston.

[CIT0011] Kongkhiaw C 2010 Video system for dynamic motion analysis of human gait. [master’s thesis]. Thailand (TH): Prince of Songkla University.

[CIT0012] Kwon JW, Son SM, Lee NK 2015 Changes of kinematic parameters of lower extremities with gait speed: a 3D motion analysis study. J Phys Ther Sci. 27(2):477–479. 25729195 10.1589/jpts.27.477 PMC4339165

[CIT0013] Macionis V 2013 Reliability of the standard goniometry and diagrammatic recording of finger joint angles: a comparative study with healthy subjects and non-professional raters. BMC Musculoskelet Disord. 14:17. 23302419 10.1186/1471-2474-14-17 PMC3557198

[CIT0014] McDowell BC, Hewitt V, Nurse A, Weston T, Baker R 2000 The variability of goniometric measurements in ambulatory children with spastic cerebral palsy. Gait Posture. 12(2):114–121. 10998607 10.1016/s0966-6362(00)00068-0

[CIT0015] McGinley JL, Baker R, Wolfe R, Morris ME 2009 The reliability of three-dimensional kinematic gait measurements: a systematic review. Gait Posture. 29(3):360–369. 19013070 10.1016/j.gaitpost.2008.09.003

[CIT0016] Miller A, Callister R 2009 Reliable lower limb musculoskeletal profiling using easily operated, portable equipment. Phys Ther Sport. 10(1):30–37. 19218077 10.1016/j.ptsp.2008.10.003

[CIT0017] Nordin M, Frankel VH 2012 Basic biomechanics of the musculoskeletal system. 4th ed. Philadelphia (PA): Lippincott Williams & Wilkins.

[CIT0018] Portney LG, Watkins MP 2009 Foundations of clinical research: applications to practice. 3rd ed. New Jersey (NJ): Prentice Hall.

[CIT0019] Simon S 2004 Quantification of human motion: gait analysis-benefits and limitations to its application to clinical problems. J Biomech. 37(12):1869–1880. 15519595 10.1016/j.jbiomech.2004.02.047

[CIT0020] Wang F, Stone E, Skubic M, Keller JM, Abbott C, Rantz M 2013 Towards a passive low-cost in-home gait assessment system for older adults. IEEE J Biomed Health Inform. 17(2):346–355. 24235111 10.1109/JBHI.2012.2233745 PMC3831173

[CIT0021] Watelain E, Froger J, Rousseaux M, Lensel G, Barbier F, Lepoutre FX, Thevenon A 2005 Variability of video-based clinical gait analysis in hemiplegia as performed by practitioners in diverse specialties. J Rehabil Med. 37(5):317–324. 16208865 10.1080/16501970510035610

[CIT0022] Williams G, Morris ME, Schache A, McCrory P 2009 Observational gait analysis in traumatic brain injury: accuracy of clinical judgment. Gait Posture. 29(3):454–459. 19109020 10.1016/j.gaitpost.2008.11.005

