# Supplementary material for: Reliability of a low-cost webcam recording system for three-dimensional lower limb gait analysis
Source: Int Biomech. 2019 Sep 26;6(1):85–92. doi: 10.1080/23335432.2019.1671221 (PMC7857306; doi:10.1080/23335432.2019.1671221)
Supplement: Supplemental Material [file TBBE_A_1671221_SM4664.doc]

**Table S1: The joint angles** (mean ± SD) obtained by the 3D-WCRS and standard motion analysis system (sagittal plane); n = 20

| **phases** | **hip joint^a^** | | | | | | **knee joint^b^** | | | | | | **ankle joint^c^** | | | | | | |
| --- | --- | --- | --- | --- | --- | --- | --- | --- | --- | --- | --- | --- | --- | --- | --- | --- | --- | --- | --- |
|  | **3D-WCRS** | | | | **standard system** | | **3D-WCRS** | | | | **standard system** | | **3D-WCRS** | | | | | **standard system** | |
|  | **1^st^ session** | | **2^nd^ session** | |  |  | **1^st^ session** | | **2^nd^ session** | |  |  | **1^st^ session** | | **2^nd^ session** | | |  |  |
|  | **mean** | **SD** | **mean** | **SD** | **mean** | **SD** | **mean** | **SD** | **mean** | **SD** | **mean** | **SD** | **mean** | **SD** | **mean** | **SD** | **mean** | | **SD** |
| **HS** | 27.82^o^ | 1.65^o^ | 28.65^o^ | 1.40^o^ | 28.23^o^ | 1.08^o^ | 5.91^o^ | 1.53^o^ | 6.71^o^ | 2.19^o^ | 2.92^o^ | 1.32^o^ | -3.93^o^ | 1.95^o^ | -3.85^o^ | 1.98^o^ | 1.74^o^ | | 1.04^o^ |
| **FF** | 18.67^o^ | 2.40^o^ | 18.81^o^ | 2.27^o^ | 23.66^o^ | 1.44^o^ | 9.59^o^ | 1.95^o^ | 9.59^o^ | 2.90^o^ | 8.71^o^ | 1.89^o^ | -5.30^o^ | 2.28^o^ | -5.88^o^ | 1.74^o^ | -3.47^o^ | | 1.10^o^ |
| **MS** | -0.90^o^ | 1.46^o^ | -0.49^o^ | 1.40^o^ | -2.60^o^ | 1.50^o^ | 5.54^o^ | 1.32^o^ | 6.11^o^ | 1.52^o^ | 1.09^o^ | 1.70^o^ | 7.99^o^ | 1.08^o^ | 7.04^o^ | 1.10^o^ | 9.49^o^ | | 1.21^o^ |
| **HO** | -11.63^o^ | 0.89^o^ | -11.24^o^ | 1.10^o^ | -15.62^o^ | 0.78^o^ | 12.07^o^ | 2.06^o^ | 13.28^o^ | 2.14^o^ | 5.11^o^ | 1.39^o^ | 10.90^o^ | 1.49^o^ | 10.15^o^ | 1.45^o^ | 11.93^o^ | | 1.68^o^ |
| **TO** | -7.31^o^ | 2.37^o^ | -6.80^o^ | 2.15^o^ | -13.28^o^ | 1.28^o^ | 33.22^o^ | 5.09^o^ | 34.35^o^ | 4.03^o^ | 23.94^o^ | 2.82^o^ | -10.95^o^ | 5.76^o^ | -11.90^o^ | 5.16^o^ | -11.26^o^ | | 3.05^o^ |
| **ISW** | 12.92^o^ | 2.62^o^ | 14.22^o^ | 2.43^o^ | 9.03^o^ | 1.48^o^ | 57.75^o^ | 2.56^o^ | 58.76^o^ | 2.65^o^ | 52.41^o^ | 1.82^o^ | -5.36^o^ | 3.49^o^ | -5.40^o^ | 3.43^o^ | -7.61^o^ | | 2.50^o^ |
| **MSW** | 23.96^o^ | 1.23^o^ | 24.43^o^ | 1.27^o^ | 24.41^o^ | 1.31^o^ | 31.50^o^ | 4.87^o^ | 30.93^o^ | 5.42^o^ | 34.31^o^ | 2.21^o^ | 0.70^o^ | 1.39^o^ | -0.11^o^ | 1.63^o^ | 7.19^o^ | | 1.04^o^ |
| **LSW** | 27.75^o^ | 1.55^o^ | 28.05^o^ | 1.39^o^ | 28.37^o^ | 1.26^o^ | 7.05^o^ | 2.23^o^ | 7.14^o^ | 1.80^o^ | 4.53^o^ | 1.55^o^ | -5.52^o^ | 1.18^o^ | -6.22^o^ | 1.31^o^ | 2.77^o^ | | 1.14^o^ |

3D-WCRS: three dimensional gait analysis of hip, knee and ankle joints, HS: heel strike; FF: foot flat; MS: midstance; HO: heel off; TO: toe off; ISW: initial swing; MSW: mid swing; LSW: late swing

a: positive values (+) and negative values (-) were hip flexion hip extension and extension, respectively

b: positive values (+) and negative values (-) were knee flexion and knee extension, respectively

c: positive values (+) and negative values (-) were ankle dorsiflexion and ankle plantarflexion, respectively

**Table S2: The joint angles** (mean ± SD) obtained by the 3D-WCRS and standard motion analysis system (frontal plane); n = 20

| **phases** | **hip joint^a^** | | | | | | **knee joint^b^** | | | | | | **ankle joint^c^** | | | | | |
| --- | --- | --- | --- | --- | --- | --- | --- | --- | --- | --- | --- | --- | --- | --- | --- | --- | --- | --- |
|  | **3D-WCRS** | | | | **standard system** | | **3D-WCRS** | | | | **standard system** | | **3D-WCRS** | | | | **standard system** | |
|  | **1^st^ session** | | **2^nd^ session** | |  |  | **1^st^ session** | | **2^nd^ session** | |  |  | **1^st^ session** | | **2^nd^ session** | |  |  |
|  | **mean** | **SD** | **mean** | **SD** | **mean** | **SD** | **mean** | **SD** | **mean** | **SD** | **mean** | **SD** | **mean** | **SD** | **mean** | **SD** | **mean** | **SD** |
| **HS** | 3.30^o^ | 1.92^o^ | 3.57^o^ | 1.83^o^ | 0.76^o^ | 1.04^o^ | -1.84^o^ | 0.80^o^ | -2.12^o^ | 1.70^o^ | -2.00^o^ | 0.39^o^ | 1.89^o^ | 7.66^o^ | 2.71^o^ | 7.76^o^ | 0.23^o^ | 0.36^o^ |
| **FF** | 2.77^o^ | 2.14^o^ | 2.91^o^ | 2.24^o^ | 5.50^o^ | 1.13^o^ | 0.08^o^ | 0.92^o^ | -0.22^o^ | 1.86^o^ | -0.11^o^ | 0.72^o^ | -0.24^o^ | 3.82^o^ | -1.50^o^ | 4.42^o^ | 2.96^o^ | 0.51^o^ |
| **MS** | 3.04^o^ | 1.48^o^ | 3.38^o^ | 1.54^o^ | 4.88^o^ | 0.48^o^ | 0.52^o^ | 0.33^o^ | 0.32^o^ | 1.02^o^ | -1.56^o^ | 0.32^o^ | 2.14^o^ | 3.77^o^ | 1.08^o^ | 3.59^o^ | 2.06^o^ | 0.45^o^ |
| **HO** | 2.96^o^ | 1.06^o^ | 3.13^o^ | 1.12^o^ | 2.77^o^ | 0.54^o^ | 0.06^o^ | 1.17^o^ | -0.11^o^ | 2.79^o^ | -1.47^o^ | 0.33^o^ | 0.00^o^ | 2.76^o^ | -0.36^o^ | 2.68^o^ | 0.82^o^ | 0.43^o^ |
| **TO** | -1.76^o^ | 2.45^o^ | -1.6 3^o^ | 2.44^o^ | -3.87^o^ | 1.22^o^ | 5.29^o^ | 2.21^o^ | 5.59^o^ | 5.59^o^ | 6.32^o^ | 2.00^o^ | -2.83^o^ | 5.02^o^ | -3.27^o^ | 5.22^o^ | 1.19^o^ | 0.69^o^ |
| **ISW** | -1.81^o^ | 2.90^o^ | -1.77^o^ | 2.94^o^ | -5.48^o^ | 0.61^o^ | 7.78^o^ | 1.29^o^ | 7.40^o^ | 2.75^o^ | 20.31^o^ | 1.35^o^ | 10.26^o^ | 4.10^o^ | 9.45^o^ | 4.27^o^ | 5.43^o^ | 0.38^o^ |
| **MSW** | 1.32^o^ | 1.59^o^ | 1.13^o^ | 1.24^o^ | -0.01^o^ | 0.70^o^ | -0.49^o^ | 2.39^o^ | -2.68^o^ | 7.69^o^ | 16.56^o^ | 1.45^o^ | 10.95^o^ | 3.38^o^ | 11.21^o^ | 4.79^o^ | 6.01^o^ | 0.38^o^ |
| **LSW** | 2.87^o^ | 1.46^o^ | 2.71^o^ | 1.44^o^ | 0.71^o^ | 0.73^o^ | -3.17^o^ | 0.98^o^ | -3.21^o^ | 2.58^o^ | -1.78^o^ | 0.60^o^ | 6.07^o^ | 5.30^o^ | 5.59^o^ | 6.14^o^ | 0.63^o^ | 0.50^o^ |

3D-WCRS: three dimensional gait analysis of hip, knee and ankle joints, HS: heel strike; FF: foot flat; MS: midstance; HO: heel off; TO: toe off; ISW: initial swing; MSW: mid swing; LSW: late swing

a: positive values (+) and negative values (-) were hip flexion hip extension and extension, respectively

b: positive values (+) and negative values (-) were knee flexion and knee extension, respectively

c: positive values (+) and negative values (-) were ankle dorsiflexion and ankle plantarflexion, respectively

**Table S3: The joint angles** (mean ± SD) obtained by the 3D-WCRS and standard motion analysis system (horizontal plane); n =20

| **phases** | **hip joint^a^** | | | | | | **knee joint^b^** | | | | | | **ankle joint^c^** | | | | | |
| --- | --- | --- | --- | --- | --- | --- | --- | --- | --- | --- | --- | --- | --- | --- | --- | --- | --- | --- |
|  | **3D-WCRS** | | | | **standard system** | | **3D-WCRS** | | | | **standard system** | | **3D-WCRS** | | | | **standard system** | |
|  | **1^st^ session** | | **2^nd^ session** | |  |  | **1^st^ session** | | **2^nd^ session** | |  |  | **1^st^ session** | | **2^nd^ session** | |  |  |
|  | **mean** | **SD** | **mean** | **SD** | **mean** | **SD** | **mean** | **SD** | **mean** | **SD** | **mean** | **SD** | **mean** | **SD** | **mean** | **SD** | **mean** | **SD** |
| **HS** | -2.93^o^ | 3.81^o^ | -2.86^o^ | 4.47^o^ | -6.49^o^ | 2.46^o^ | 4.19^o^ | 4.08^o^ | 5.16^o^ | 4.20^o^ | -4.67^o^ | 1.65^o^ | 3.74^o^ | 2.43^o^ | 2.86^o^ | 2.32^o^ | -2.70^o^ | 1.59^o^ |
| **FF** | 7.54^o^ | 4.20^o^ | 8.50^o^ | 4.06^o^ | 8.21^o^ | 2.56^o^ | 4.79^o^ | 4.56^o^ | 2.93^o^ | 5.10^o^ | -4.40^o^ | 1.46^o^ | 1.75^o^ | 2.15^o^ | 1.33^o^ | 2.10^o^ | -14.68^o^ | 2.15^o^ |
| **MS** | 3.19^o^ | 2.17^o^ | 3.87^o^ | 2.20^o^ | 8.23^o^ | 1.69^o^ | 3.92^o^ | 2.99^o^ | 1.53^o^ | 3.16^o^ | -6.31^o^ | 1.54^o^ | 0.94^o^ | 2.10^o^ | 0.19^o^ | 1.87^o^ | -10.85^o^ | 1.92^o^ |
| **HO** | -2.0 7^o^ | 2.09^o^ | -1.8 8^o^ | 1.70^o^ | 3.44^o^ | 0.96^o^ | 8.03^o^ | 2.38^o^ | 6.47^o^ | 2.51^o^ | -3.86^o^ | 1.23^o^ | -0.9 1^o^ | 2.30^o^ | -1.81^o^ | 2.42^o^ | -5.27^o^ | 1.95^o^ |
| **TO** | -2.61^o^ | 2.46^o^ | -2.34^o^ | 2.90^o^ | 9.94^o^ | 1.95^o^ | 3.45^o^ | 3.68^o^ | 2.55^o^ | 3.70^o^ | -7.43^o^ | 1.37^o^ | -0.19^o^ | 7.53^o^ | -0.27^o^ | 7.80^o^ | -6.90^o^ | 3.16^o^ |
| **ISW** | -0.57^o^ | 2.49^o^ | -1.13^o^ | 2.00^o^ | 19.30^o^ | 1.53^o^ | 4.10^o^ | 3.87^o^ | 4.64^o^ | 4.35^o^ | -3.79^o^ | 1.71^o^ | -15.92^o^ | 5.25^o^ | -15.40^o^ | 5.22^o^ | -24.57^o^ | 1.49^o^ |
| **MSW** | 6.55^o^ | 2.83^o^ | 7.02^o^ | 3.05^o^ | 21.70^o^ | 2.06^o^ | -13.24^o^ | 4.94^o^ | -15.12^o^ | 5.10^o^ | -10.10^o^ | 1.58^o^ | -11.22^o^ | 3.74^o^ | -11.23^o^ | 4.18^o^ | -26.58^o^ | 1.36^o^ |
| **LSW** | -5.10^o^ | 3.70^o^ | -7.23^o^ | 4.13^o^ | -5.24^o^ | 2.98^o^ | -0.45^o^ | 4.76^o^ | 1.84^o^ | 4.50^o^ | -5.09^o^ | 1.83^o^ | -1.95^o^ | 2.39^o^ | -1.79^o^ | 2.44^o^ | -3.17^o^ | 2.27^o^ |

3D-WCRS: three dimensional gait analysis of hip, knee and ankle joints, HS: heel strike; FF: foot flat; MS: midstance; HO: heel off; TO: toe off; ISW: initial swing; MSW: mid swing; LSW: late swing

a: positive values (+) and negative values (-) were hip flexion hip extension and extension, respectively

b: positive values (+) and negative values (-) were knee flexion and knee extension, respectively

c: positive values (+) and negative values (-) were ankle dorsiflexion and ankle plantarflexion, respectively

**Table S4:** **Means and standard errors of measurement (SEM) obtained by 3D-WCRS and standard motion analysis system of one gait cycle in sagittal plane (n = 20)**

| **phases** | **hip joint^a^** | | | | | | **knee joint^b^** | | | | | | **ankle joint^c^** | | | | | |
| --- | --- | --- | --- | --- | --- | --- | --- | --- | --- | --- | --- | --- | --- | --- | --- | --- | --- | --- |
|  | **3D-WCRS** | | | | **standard system** | | **3D-WCRS** | | | | **standard system** | | **3D-WCRS** | | | | **standard system** | |
|  | **1^st^ session** | | **2^nd^ session** | |  |  | **1^st^ session** | | **2^nd^ session** | |  |  | **1^st^ session** | | **2^nd^ session** | |  |  |
|  | **mean** | **SEM** | **mean** | **SEM** | **mean** | **SEM** | **mean** | **SEM** | **mean** | **SEM** | **mean** | **SEM** | **mean** | **SEM** | **mean** | **SEM** | **mean** | **SEM** |
| **HS** | 27.82^o^ | 0.29 ^o^ | 28.65 ^o^ | 0.20 ^o^ | 28.23 ^o^ | 0.11 ^o^ | 5.91 ^o^ | 0.22 ^o^ | 6.71 ^o^ | 0.76 ^o^ | 2.92 ^o^ | 0.19 ^o^ | -3.93 ^o^ | 0.20 ^o^ | -3.85 ^o^ | 0.52 ^o^ | 1.74 ^o^ | 0.18 ^o^ |
| **FF** | 18.67 ^o^ | 0.48 ^o^ | 18.81 ^o^ | 0.45 ^o^ | 23.66 ^o^ | 0.14 ^o^ | 9.59 ^o^ | 0.28 ^o^ | 9.59 ^o^ | 0.82 ^o^ | 8.71 ^o^ | 0.38 ^o^ | -5.30 ^o^ | 0.39 ^o^ | -5.88 ^o^ | 0.39 ^o^ | -3.47 ^o^ | 0.19 ^o^ |
| **MS** | -0.90 ^o^ | 0.25 ^o^ | -0.49 ^o^ | 0.24 ^o^ | -2.60 ^o^ | 0.34 ^o^ | 5.54 ^o^ | 0.19 ^o^ | 6.11 ^o^ | 0.21 ^o^ | 1.09 ^o^ | 0.42 ^o^ | 7.99 ^o^ | 0.11 ^o^ | 7.04 ^o^ | 0.22 ^o^ | 9.49 ^o^ | 0.27 ^o^ |
| **HO** | -11.63 ^o^ | 0.09 ^o^ | -11.24 ^o^ | 0.16 ^o^ | -15.62 ^o^ | 0.08 ^o^ | 12.07 ^o^ | 0.46 ^o^ | 13.28 ^o^ | 0.43 ^o^ | 5.11 ^o^ | 0.14 ^o^ | 10.90 ^o^ | 0.15 ^o^ | 10.15 ^o^ | 0.36 ^o^ | 11.93 ^o^ | 0.29 ^o^ |
| **TO** | -7.31 ^o^ | 0.63 ^o^ | -6.80 ^o^ | 0.61 ^o^ | -13.28 ^o^ | 0.13 ^o^ | 33.22 ^o^ | 2.33 ^o^ | 34.35 ^o^ | 1.93 ^o^ | 23.94 ^o^ | 0.63 ^o^ | -10.95 ^o^ | 1.52 ^o^ | -11.90 ^o^ | 2.31 ^o^ | -11.26 ^o^ | 0.86 ^o^ |
| **ISW** | 12.92 ^o^ | 0.83 ^o^ | 14.22 ^o^ | 0.73 ^o^ | 9.03 ^o^ | 0.21 ^o^ | 57.75 ^o^ | 0.63 ^o^ | 58.76 ^o^ | 0.75 ^o^ | 52.41 ^o^ | 0.18 ^o^ | -5.36 ^o^ | 0.70 ^o^ | -5.40 ^o^ | 1.41 ^o^ | -7.61 ^o^ | 0.65 ^o^ |
| **MSW** | 23.96 ^o^ | 0.17 ^o^ | 24.43 ^o^ | 0.18 ^o^ | 24.41 ^o^ | 0.13 ^o^ | 31.50 ^o^ | 2.07 ^o^ | 30.93 ^o^ | 2.76 ^o^ | 34.31 ^o^ | 0.22 ^o^ | 0.70 ^o^ | 0.14 ^o^ | -0.11 ^o^ | 0.49 ^o^ | 7.19 ^o^ | 0.25 ^o^ |
| **LSW** | 27.75 ^o^ | 0.27 ^o^ | 28.05 ^o^ | 0.24 ^o^ | 28.37 ^o^ | 0.13 ^o^ | 7.05 ^o^ | 0.54 ^o^ | 7.14 ^o^ | 0.31 ^o^ | 4.53 ^o^ | 0.31 ^o^ | -5.52 ^o^ | 0.12 ^o^ | -6.22 ^o^ | 0.23 ^o^ | 2.77 ^o^ | 0.20 ^o^ |

3D-WCRS: three dimensional gait analysis of hip, knee and ankle joints, HS: heel strike; FF: foot flat; MS: midstance; HO: heel off; TO: toe off; ISW: initial swing; MSW: mid swing; LSW: late swing

a: positive values (+) and negative values (-) were hip flexion hip extension and extension, respectively

b: positive values (+) and negative values (-) were knee flexion and knee extension, respectively

c: positive values (+) and negative values (-) were ankle dorsiflexion and ankle plantarflexion, respectively

**Table S5: Means and standard errors of measurement (SEM) obtained by 3D-WCRS and Vicon^TM^** of one gait cycle in frontal plane (n = 20)

| **phases** | **hip joint^a^** | | | | | | **knee joint^b^** | | | | | | **ankle joint^c^** | | | | | |
| --- | --- | --- | --- | --- | --- | --- | --- | --- | --- | --- | --- | --- | --- | --- | --- | --- | --- | --- |
|  | **3D-WCRS** | | | | **standard system** | | **3D-WCRS** | | | | **standard system** | | **3D-WCRS** | | | | **standard system** | |
|  | **1^st^ session** | | **2^nd^ session** | |  |  | **1^st^ session** | | **2^nd^ session** | |  |  | **1^st^ session** | | **2^nd^ session** | |  |  |
|  | **mean** | **SEM** | **mean** | **SEM** | **mean** | **SEM** | **mean** | **SEM** | **mean** | **SEM** | **mean** | **SEM** | **mean** | **SEM** | **mean** | **SEM** | **mean** | **SEM** |
| **HS** | 3.30 ^o^ | 0.74 ^o^ | 3.57 ^o^ | 0.64 ^o^ | 0.76 ^o^ | 0.15 ^o^ | -1.84 ^o^ | 0.37 ^o^ | -2.12 ^o^ | 0.96 ^o^ | -2.00 ^o^ | 0.04 ^o^ | 1.89 ^o^ | 2.03 ^o^ | 2.71 ^o^ | 1.90 ^o^ | 0.23 ^o^ | 0.04 ^o^ |
| **FF** | 2.77 ^o^ | 0.93 ^o^ | 2.91 ^o^ | 1.19 ^o^ | 5.50 ^o^ | 0.20 ^o^ | 0.08 ^o^ | 0.29 ^o^ | -0.22 ^o^ | 0.72 ^o^ | -0.11 ^o^ | 0.07 ^o^ | -0.24 ^o^ | 0.76 ^o^ | -1.50 ^o^ | 0.88 ^o^ | 2.96 ^o^ | 0.05 ^o^ |
| **MS** | 3.04 ^o^ | 0.61 ^o^ | 3.38 ^o^ | 0.56 ^o^ | 4.88 ^o^ | 0.05 ^o^ | 0.52 ^o^ | 0.08 ^o^ | 0.32 ^o^ | 0.23 ^o^ | -1.56 ^o^ | 0.03 ^o^ | 2.14 ^o^ | 0.75 ^o^ | 1.08 ^o^ | 0.72 ^o^ | 2.06 ^o^ | 0.05 ^o^ |
| **HO** | 2.96 ^o^ | 0.28 ^o^ | 3.13 ^o^ | 0.25 ^o^ | 2.77 ^o^ | 0.05 ^o^ | 0.06 ^o^ | 0.45 ^o^ | -0.11 ^o^ | 1.37 ^o^ | -1.47 ^o^ | 0.03 ^o^ | 0.00 ^o^ | 0.39 ^o^ | -0.36 ^o^ | 0.38 ^o^ | 0.82 ^o^ | 0.04 ^o^ |
| **TO** | -1.76 ^o^ | 1.10 ^o^ | -1.63 ^o^ | 0.98 ^o^ | -3.87 ^o^ | 0.21 ^o^ | 5.29 ^o^ | 1.29 ^o^ | 5.59 ^o^ | 3.16 ^o^ | 6.32 ^o^ | 0.28 ^o^ | -2.83 ^o^ | 1.42 ^o^ | -3.27 ^o^ | 1.28 ^o^ | 1.19 ^o^ | 0.07 ^o^ |
| **ISW** | -1.81 ^o^ | 1.42 ^o^ | -1.77 ^o^ | 1.50 ^o^ | -5.48 ^o^ | 0.06 ^o^ | 7.78 ^o^ | 0.36 ^o^ | 7.40 ^o^ | 0.67 ^o^ | 20.31 ^o^ | 0.14 ^o^ | 10.26 ^o^ | 0.58 ^o^ | 9.45 ^o^ | 0.60 ^o^ | 5.43 ^o^ | 0.04 ^o^ |
| **MSW** | 1.32 ^o^ | 0.59 ^o^ | 1.13 ^o^ | 0.28 ^o^ | -0.01 ^o^ | 0.07 ^o^ | -0.49 ^o^ | 1.33 ^o^ | -2.68 ^o^ | 5.60 ^o^ | 16.56 ^o^ | 0.15 ^o^ | 10.95 ^o^ | 0.48 ^o^ | 11.21 ^o^ | 0.83 ^o^ | 6.01 ^o^ | 0.05 ^o^ |
| **LSW** | 2.87 ^o^ | 0.44 ^o^ | 2.71 ^o^ | 0.35 ^o^ | 0.71 ^o^ | 0.07 ^o^ | -3.17 ^o^ | 0.65 ^o^ | -3.21 ^o^ | 1.77 ^o^ | -1.78 ^o^ | 0.06 ^o^ | 6.07 ^o^ | 1.06 ^o^ | 5.59 ^o^ | 0.87 ^o^ | 0.63 ^o^ | 0.05 ^o^ |

3D-WCRS: three dimensional gait analysis of hip, knee and ankle joints, HS: heel strike; FF: foot flat; MS: midstance; HO: heel off; TO: toe off; ISW: initial swing; MSW: mid swing; LSW: late swing

a: positive values (+) and negative values (-) were hip adduction and hip abduction

b: positive values (+) and negative values (-) were knee adduction and knee abduction

c: positive values (+) and negative values (-) were ankle inversion and ankle eversion

**Table S6:** **Means and standard errors of measurement (SEM) obtained by 3D-WCRS and Vicon^TM^ of one gait cycle in horizontal plane (n = 20)**

| **phases** | **hip joint^a^** | | | | | | **knee joint^b^** | | | | | | **ankle joint^c^** | | | | | |
| --- | --- | --- | --- | --- | --- | --- | --- | --- | --- | --- | --- | --- | --- | --- | --- | --- | --- | --- |
|  | **3D-WCRS** | | | | **standard system** | | **3D-WCRS** | | | | **standard system** | | **3D-WCRS** | | | | **standard system** | |
|  | **1^st^ session** | | **2^nd^ session** | |  |  | **1^st^ session** | | **2^nd^ session** | |  |  | **1^st^ session** | | **2^nd^ session** | |  |  |
|  | **mean** | **SEM** | **mean** | **SEM** | **mean** | **SEM** | **mean** | **SEM** | **mean** | **SEM** | **mean** | **SEM** | **mean** | **SEM** | **mean** | **SEM** | **mean** | **SEM** |
| **HS** | -2.93 ^o^ | 1.01 ^o^ | -2.86 ^o^ | 1.48 ^o^ | -6.49 ^o^ | 0.25 ^o^ | 4.19 ^o^ | 1.29 ^o^ | 5.16 ^o^ | 1.03 ^o^ | -4.67 ^o^ | 0.17 ^o^ | 3.74 ^o^ | 0.54 ^o^ | 2.86 ^o^ | 0.33 ^o^ | -2.70 ^o^ | 0.16 ^o^ |
| **FF** | 7.54 ^o^ | 1.45 ^o^ | 8.50 ^o^ | 1.15 ^o^ | 8.21 ^o^ | 0.26 ^o^ | 4.79 ^o^ | 2.99 ^o^ | 2.93 ^o^ | 1.98 ^o^ | -4.40 ^o^ | 0.15 ^o^ | 1.75 ^o^ | 0.30 ^o^ | 1.33 ^o^ | 0.30 ^o^ | -14.68 ^o^ | 0.22 ^o^ |
| **MS** | 3.19 ^o^ | 0.49 ^o^ | 3.87 ^o^ | 0.44 ^o^ | 8.23 ^o^ | 0.17 ^o^ | 3.92 ^o^ | 1.16 ^o^ | 1.53 ^o^ | 0.89 ^o^ | -6.31 ^o^ | 0.15 ^o^ | 0.94 ^o^ | 0.30 ^o^ | 0.19 ^o^ | 0.26 ^o^ | -10.85 ^o^ | 0.19 ^o^ |
| **HO** | -2.07 ^o^ | 0.36 ^o^ | -1.88 ^o^ | 0.24 ^o^ | 3.44 ^o^ | 0.10 ^o^ | 8.03 ^o^ | 0.67 ^o^ | 6.47 ^o^ | 0.43 ^o^ | -3.86 ^o^ | 0.12 ^o^ | -0.91 ^o^ | 0.33 ^o^ | -1.81 ^o^ | 0.34 ^o^ | -5.27 ^o^ | 0.20 ^o^ |
| **TO** | -2.61 ^o^ | 0.43 ^o^ | -2.34 ^o^ | 0.50 ^o^ | 9.94 ^o^ | 0.20 ^o^ | 3.45 ^o^ | 0.82 ^o^ | 2.55 ^o^ | 0.83 ^o^ | -7.43 ^o^ | 0.14 ^o^ | -0.19 ^o^ | 3.10 ^o^ | -0.27 ^o^ | 1.91 ^o^ | -6.90 ^o^ | 0.45 ^o^ |
| **ISW** | -0.57 ^o^ | 0.35 ^o^ | -1.13 ^o^ | 0.20 ^o^ | 19.30 ^o^ | 0.15 ^o^ | 4.10 ^o^ | 0.55 ^o^ | 4.64 ^o^ | 1.38 ^o^ | -3.79 ^o^ | 0.17 ^o^ | -15.92 ^o^ | 1.17 ^o^ | -15.40 ^o^ | 1.17 ^o^ | -24.57 ^o^ | 0.15 ^o^ |
| **MSW** | 6.55 ^o^ | 0.53 ^o^ | 7.02 ^o^ | 0.61 ^o^ | 21.70 ^o^ | 0.11 ^o^ | -13.24 ^o^ | 1.48 ^o^ | -15.12 ^o^ | 1.14 ^o^ | -10.10 ^o^ | 0.16 ^o^ | -11.22 ^o^ | 0.53 ^o^ | -11.23 ^o^ | 0.72 ^o^ | -26.58 ^o^ | 0.14 ^o^ |
| **LSW** | -5.10 ^o^ | 0.91 ^o^ | -7.23 ^o^ | 0.92 ^o^ | -5.24 ^o^ | 0.30 ^o^ | -0.45 ^o^ | 1.17 ^o^ | 1.84 ^o^ | 0.90 ^o^ | -5.09 ^o^ | 0.18 ^o^ | -1.95 ^o^ | 0.24 ^o^ | -1.79 ^o^ | 0.35 ^o^ | -3.17 ^o^ | 0.23 ^o^ |

3D-WCRS: three dimensional gait analysis of hip, knee and ankle joints, HS: heel strike; FF: foot flat; MS: midstance; HO: heel off; TO: toe off; ISW: initial swing; MSW: mid swing; LSW: late swing

a: positive values (+) and negative values (-) were hip internal rotation and hip external rotation

b: positive values (+) and negative values (-) were knee internal rotation and knee external rotation

c: positive values (+) and negative values (-) were ankle internal rotation and ankle external rotation
